# How Is COVID-19 Affecting South Korea? What Is Our Current Strategy?

**DOI:** 10.1017/dmp.2020.69

**Published:** 2020-04-03

**Authors:** Minyoung Her

**Affiliations:** Department of Internal Medicine, Pyeongtaek Good morning Hospital, Pyeongtaek, South Korea

**Keywords:** COVID-19, outbreak, South Korea

## Abstract

The outbreak of coronavirus disease 2019 (COVID-19) caused by the virus SARS-CoV-2 is expanding globally. South Korea is one of the countries most affected by COVID-19 from the very early stages of this pandemic. Explosive outbreaks occurred across South Korea in the first two months, and efforts to control this new virus have involved everyone across the country. To curb the transmission of the virus, health-care professionals, committees, and governments have combined many approaches, such as extensive COVID-19 screening, effective patient triage, the transparent provision of information, and the use of information technology. This experience could provide some valuable ideas and lessons to others who are fighting against COVID-19.

Since the outbreak of coronavirus disease 2019 (COVID-19) caused by the virus SARS-CoV-2 started in Wuhan, China, at the end of December 2019, there has been a great deal of chaos.^1^ Although South Korea is one of the closest countries to China, we were not fully alerted about the need for precautions and extensive preparedness with regard to this newly emerging virus until mid-February.

However, in February, in South Korea and especially in Daegu city, local transmission was identified, multiple clusters were confirmed, and the situation was transformed. Various approaches, such as extensive COVID-19 screening, effective patient triage, the transparent disclosure of information and the use of information technology, were introduced to stop the transmission of the virus.

In this study, we discuss how South Korea was strongly hit by COVID-19 from the very early stages of the pandemic and the strategies used to combat the outbreak.

## STRONG IMPACT OF COVID-19 ON SOUTH KOREA

The first case of COVID-19 in South Korea, a female who was from Wuhan city and stopped over in the South Korean airport, was reported in January 2020. Until more than 30 cases of COVID-19 occurred in Korea, the situation seemed to be under control. There was a suggestion from the health-care committee to strengthen border control and disinfection protocols, based on concern regarding the contagiousness of COVID-19; however, this recommendation was dismissed.

In mid-February, there was a turning point that led to an explosive outbreak in Korea. A person (31st case) who developed atypical pneumonia without any history of travel to an area with a COVID-19 outbreak was identified in the Daegu area, and the turmoil began.^2^ Following this case, hundreds of new patients were reported every day, especially in Daegu and the neighboring area. A religious gathering was found to be the hot spot at the center of the spread of the virus. Around that time in the neighboring area, a substantial portion of the patients in closed neuropsychiatric wards developed respiratory symptoms. Later, more than 100 people, including most patients and some health professionals in the ward, were diagnosed with COVID-19. Chinese caregivers who had visited Wuhan and subsequently volunteered in a religious capacity in this hospital were presumed to be the origin of the outbreak in Daegu, although the epidemiological connection was still under-investigated. Many new clusters were reported in hospitals, senior care facilities, indoor sports facilities, banquets, and workplaces, such as call centers. According to the Korean Centers for Disease Control and Prevention, more than 80% of the COVID-19 cases were related to these clusters.

In Daegu and the neighboring areas, the outbreaks were not controlled, and most hospitals became full of COVID-19 patients. In March 2020, there were more than 7000 patients in this area, and even confirmed patients were not hospitalized. There were patients, especially elderly patients, who had many comorbidities and died while waiting for admission. Among the more than 9000 patients with COVID-19 in South Korea, 158 patients died. More than 90% of the patients were from Daegu and the surrounding areas.^2^

There was also a serious shortage of health-care staff and protective equipment. The government recruited volunteer health-care professionals, including physicians and nurses, and dispatched military medical teams to address the outbreaks in Daegu, but these measures were not enough. These strategies did increase the speed of patient triage, enabling critical patients to be treated faster and decreasing the mortality rate. Patients were classified based on COVID-19 severity determined by the modified early warning score^3^ and several other factors. The categories were mild, moderate, severe, and critical. Using this triage system, the treatment location was determined for each patient. For example, patients with mild COVID-19, especially those younger than 50 years old, were instructed to stay in a designated place with monitoring or to remain at home instead of in the hospital. However, sometimes this system did not function adequately. In some cases, patients with mild cases who were already occupying hospital beds refused to move to another location because they insisted that it was first come, first served, regardless of the patient triage system. Such a system truly needs to be applied from the beginning of an outbreak.

Having patients with mild cases remain at home poses an immediate threat of exposure to the other members of the household, and there is the possibility of quarantine violations. Therefore, it might be better for even patients with mild cases to stay in designated areas under monitoring if there are available facilities and health-care services.

## COPING WITH COVID-19 IN SOUTH KOREA

Neither a vaccine nor target treatments are available for COVID-19, and the virus is highly contagious. More importantly, there are asymptomatic patients who are able to transmit the virus.^4^ Thus, early COVID-19 screening is the key to curbing transmission. In South Korea, more than 600 COVID-19 screening sites were established, including public health-care clinics, drive-through centers, and walk-in screening sites, all of which were capable of performing COVID-19 screening and taking swab samples for reverse transcription-polymerase chain reaction (RT-PCR) tests of SARS-CoV-2 nucleic acid.^2^ Across the country, more than 15,000 screening tests could be performed in one day.

COVID-19 screening sites have several aims. One is to identify patients early to halt the spread of the virus in the community, and another is to avoid the possibility of having to shut down unaffected hospitals due to contagion. All patients who visited the hospital regardless of the reason for their visit were expected to fill out a questionnaire before entering the hospital about any fever, any respiratory symptoms, their travel history, and any history of contact with people with confirmed cases of COVID-19. Then they were allowed to enter the hospital, depending on their symptoms. The patients who were suspected of having COVID-19 were expected to visit a COVID-19 screening site first or to call the hot line before their visit.

COVID-19 screening was performed for patients who were strongly suspected of having COVID-19, as well as many asymptomatic groups with epidemiological evidence suggesting the possibility of infection. For example, all members of a specific religious group in Daegu, asymptomatic people related to COVID-19 clusters, and all pneumonia cases regardless of cause were tested for COVID-19. Those extensive screenings allowed the early identification of asymptomatic or mild patients who had viral loads. The relatively low mortality rate due to COVID-19 in South Korea might be the result of the inclusion of more asymptomatic or mild cases in the total number of cases compared with other countries.

Along with extensive screening, there are many other strategies used to address the transmission of COVID-19, including drive-through screening for COVID-19, tracking movements online with immediate alarms and notifications, developing an app for the COVID-19 screening questionnaire, and the supplying and distributing of protective gear by the public sector.

Determining the movement of patients is imperative to prevent further transmission. However, some patients did not disclose their contacts and movements. The government used Global Positioning System (GPS) records from their cellular phone or credit card records to generate a movement map. Once the movement map was made, the map was displayed on the Web or notifications were sent to inhabitants in the relevant neighborhoods so they could take additional precautions. To monitor people under quarantine, applications on smartphones using GPS data were introduced.

The drastic increase in the demand for masks or personal protective equipment led to price gouging and hoarding. The certified mask Korean filter 94, which was originally developed for filtering yellow dust, is used by most people. COVID-19 is transmitted person to person through direct contact, respiratory droplets, and aerosolized viral particles.^5,6^ Asymptomatic or mild patients who can transmit the virus could be anywhere, especially in clusters, remaining undetected and not in isolation. Keeping a social distance of more than 2 meters is not easy, especially in crowded cities. Therefore, almost everyone wore masks to prevent the spread of germs and to protect themselves regardless of any guidelines on who should be wearing masks. Because of the shortage of supplies, the government intervened in their production and distribution to deal with high needs in particular areas.

## CONCLUSIONS

In South Korea, including Daegu, routine life was completely disrupted, and the economy was severely affected. The COVID-19 outbreak is still ongoing. There are many unknown factors that affect the trajectory of this pandemic. Health-care professionals strongly insisted on applying stringent methods, such as strengthening border control, lockdowns, strong bans on gatherings, bans on exporting personal protective equipment, and stringent infection control measures from the early beginning. However, there have been many conflicts among the interests of public health, politics, religion, and the economy, and the opinions of health-care professionals were not always prioritized, even during this outbreak.

The majority of people have followed the rules and cooperated with the strategies used by the government. However, there are people who dismiss the severity of this disease, neglect the disinfection guidelines, and violate the rules. There are others who might think this is a good opportunity to make money through cornering a market and hoarding. We hope everyone abides by the rules voluntarily without the need for enforcement. However, strong penalties, fines, enforcement, legislation, and lockdowns might be more effective ways to decrease the transmission of the virus under the current conditions.

Recently, multiple clusters were reported in Seoul, the capital of South Korea, and a rapidly growing number of patients have been reported in many countries around the world. The pandemic has entered a new phase. This is not merely a battle against a virus. All our abilities, such as health-care preparedness, the cooperation of all departments, the transparency of information sharing, and the ethical standards of people, are tested in the end.
